# Identification of candidate genes for fiber length quantitative trait loci through RNA-Seq and linkage and physical mapping in cotton

**DOI:** 10.1186/s12864-017-3812-5

**Published:** 2017-05-31

**Authors:** Xihua Li, Man Wu, Guoyuan Liu, Wenfeng Pei, Honghong Zhai, Jiwen Yu, Jinfa Zhang, Shuxun Yu

**Affiliations:** 1 College of Agronomy, Northwest A&F University, Yangling, 712100 China; 2 State Key Laboratory of Cotton Biology, Institute of Cotton Research, Chinese Academy of Agricultural Science, Anyang, 455000 China; 3 Department of Plant and Environmental Sciences, New Mexico State University, Las Cruces, NM 88003 USA

**Keywords:** *Gossypium hirsutum*, *G. barbadense*, Backcross inbred lines (BILs), Fiber elongation, RNA-Seq, Single nucleotide polymorphism (SNP), Quantitative trait loci (QTL)

## Abstract

**Background:**

Cotton (*Gossypium* spp.) fibers are single-celled elongated trichomes, the molecular aspects of genetic variation in fiber length (FL) among genotypes are currently unknown. In this study, two backcross inbred lines (BILs), i.e., NMGA-062 (“Long”) and NMGA-105 (“Short”) with 32.1 vs. 27.2 mm in FL, respectively, were chosen to perform RNA-Seq on developing fibers at 10 days post anthesis (DPA). The two BILs differed in 4 quantitative trait loci (QTL) for FL and were developed from backcrosses between *G. hirsutum* as the recurrent parent and *G. barbadense*.

**Results:**

In total, 51.7 and 54.3 million reads were obtained and assembled to 49,508 and 49,448 transcripts in the two genotypes, respectively. Of 1﻿551 differentially expressed genes (DEGs) between the two BILs, 678 were up-regulated and 873 down-regulated in “Long”; and 703 SNPs were identified in 339 DEGs. Further physical mapping showed that 8 DEGs were co-localized with the 4 FL QTL identified in the BIL population containing the two BILs. Four SNP markers in 3 DEGs that showed significant correlations with FL were developed. Among the three candidate genes encoding for proline-rich protein, D-cysteine desulfhydrase, and thaumatin-like protein, a SNP of thaumatin-like protein gene showed consistent correlations with FL across all testing environments.

**Conclusions:**

This study represents one of the first investigations of positional candidate gene approach of QTL in cotton in integrating transcriptome and SNP identification based on RNA-Seq with linkage and physical mapping of QTL and genes, which will facilitate eventual cloning and identification of genes responsible for FL QTL. The candidate genes may serve as the foundation for further in-depth studies of the molecular mechanism of natural variation in fiber elongation.

**Electronic supplementary material:**

The online version of this article (doi:10.1186/s12864-017-3812-5) contains supplementary material, which is available to authorized users.

## Background

Cotton is one of the most important fiber crop and the third important oil crop in the world [1, 2]. It also serves as a model species for studies of the cell elongation process in plant biology [3].

Cotton fibers, known as the cotton lint, are single-celled trichomes that differentiate from single cells in the epidermis of the ovule. Cotton fiber development consists of four periods: fiber initiation, fiber elongation, secondary cell wall biosynthesis and maturation [4–7]. Fiber length (FL) is predominantly determined during the fiber elongation stage (5–25 days post anthesis, DPA). During the fast elongation period at 5–10 DPA, fiber cells expand vigorously, with peak growth rates of >2 mm/day in Upland cotton [8, 9], and the maximum elongation rate occurs at 10 DPA [10]. A number of studies have demonstrated that cotton fiber development is a complex biological process that involves dynamic transcriptional regulatory networks [9–25]. Fast-growing fiber cells were originally reported to expand via a tip-growth model [24] and a diffuse-growth model [6]. Recently, a combined linear cell growth model encompassing both the tip-growth model and diffuse-growth model was proposed [25]. In this model, very-long-chain fatty acids (VLCFAs) promote fiber growth by activating ethylene biosynthesis to stimulate pectin biosynthesis and scaffold establishment; and Ca^2+^, Ca^2+^-dependent protein kinase, reactive oxygen species (ROS) and sucrose synthase are also involved in fiber cell elongation [25]. However, these models do not explain the molecular mechanism underlying natural genetic differences between genotypes differing in FL.

Numerous differentially expressed genes have been identified during the fiber elongation stage in cotton using cloning and sequencing of cDNA libraries (expressed sequence tags, ESTs) and microarrays [11–15, 23]. Although many microarray-based gene expression studies have attempted to address the molecular aspects of fiber cell elongation using fiberless mutants or different species, this process remains unclear. RNA sequencing (RNA-Seq) has emerged as a high-throughput, low-cost next-generation sequencing (NGS) technique that provides a powerful technological platform to identify genes related to traits/treatments of interest, with or without genome information as a reference. Several studies regarding cotton fibers that have used NGS techniques have been published [16–19].

Although many gene expression studies on fiber development have been reported, several issues are noted here. First, most of differentially expressed genes (DEGs) identified from the comparative analysis between different species (i.e., *G. hirsutum* vs. *G. barbadense*) are related to differences between species and are unlikely to be associated with fiber development or fiber traits. Therefore, near-isogenic lines (NILs) [26] or backcross inbred lines (BILs) with the same genetic background should be used. In the case of chromosome segment introgression lines (CSILs) used [18], the numbers of RNA-Seq reads were not high enough for a comprehensive genome coverage. Second, in the case of previous studies using microarrays, one of the limitations of microarrays such as Affymetrix GeneChips is the number of probes representing genes (24,133) spotted on the GeneChip, which is far less than that predicted in the sequenced diploid and tetraploid cotton genomes [1, 27–30]. Understandably, many DEGs were not detected by microarray analysis. Third, in a few cases, the use of the predicted protein-coding gene sequences from *G. raimondii* and *G. arboreum* for gene annotation in tetraploid cotton may not be accurate enough*.* With the publication and availability of the tetraploid (AD1) genome sequence [29, 30], high-throughput NGS can identify most, if not all, of the expressed genes related to fiber quality traits including FL. Fourth, whether any of the DEGs identified from previous studies had sequence differences between a fiber mutant and its wild-type or between a CSIL and its recurrent parent is unknown. Only the DEGs that have sequence variations and also co-localize with fiber traits of interest (i.e., quantitative trait loci, QTL) are possible candidates for further studies. However, this positional candidate approach has not been used to identify genes for genetic variation in FL of cotton.

In plant breeding, molecular markers are extremely helpful for characterizing genetic variations, linkage mapping and marker-assisted selection (MAS) [31]. The narrow genetic base and allotetraploid genome nature of cotton made the discovery of SNPs difficult [32]. The use of high-throughput sequencing techniques has made it possible to detect a large number of SNP markers [33–35]. However, a genome-wide coding sequence-based SNP discovery based on RNA-Seq has not been reported using the sequenced tetraploid genome sequence.

In the past 20 years, numerous studies have reported a collection of QTL including FL QTL identified from a number of *G. hirsutum* × *G. barbadense* interspecific populations [36]. However, no candidate genes for these QTL have been identified. Currently, a genome-wide expression study of a large number of lines during fiber development is still cost prohibitive. To circumvent this problem, lines with the same genetic background such as NILs or BILs may be useful. In this study, RNA-Seq libraries were constructed and sequenced for a comparative analysis from the fast elongation fibers at 10 DPA between two BILs (NMGA-062 and NMGA-105). The two lines shared 95% of DNA markers and had similar phenotypes but a significant difference in FL (32.1 vs. 27.2 mm) and micronaire, and were selected from 146 BILs with 4 QTL for FL identified [37]. The objective of this study was to gain an understanding of molecular aspects of fiber elongation between the two BILs and to identify FL candidate genes as potential targets. The RNA-Seq depth was extremely high to cover the expressed Upland cotton genome in developing cotton fibers, and the resulting sequence reads were annotated using the published Upland cotton genome sequence as the reference [29]. Our hypothesis was that genes responsible for the genetic difference in FL (elongation) between the two BILs are among those genes that are differentially expressed in developing fibers with DNA sequence differences and co-localize with FL QTL. This study represents one of the first investigations of positional candidate gene approach of QTL in cotton in integrating transcriptome and SNP identification based on RNA-Seq with linkage and physical mapping of QTL and genes, which will facilitate the eventual cloning and identification of genes responsible for FL QTL.

## Results

### Fiber growth kinetics of the two BILs

The mature FL of “Long” (i.e., NMGA-062) and “Short” (i.e., NMGA-105) BILs averaged 32.1 and 27.2 mm, respectively, as described previously [37] (Additional file 1: Table S1) and were selected for their differences in FL and similarities in other agronomic and fiber quality traits except for micronaire. Similar to *G. barbadense*, “Long” had longer and finer (i.e., with a lower micronaire) fibers, as fiber length and fineness are often negatively correlated. Their highly similar genetic background was also evident in that they were identical in 95.4% of the SSR and SNP markers assayed (2241/2349, unpublished), which is suitable for the current comparative analysis.

**Fig. 1:**
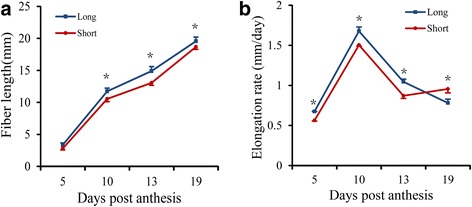
The dynamic change in fiber length during development. **a** “Long” and “Short” cotton fiber lengths at different developmental stages. **b** “Long” and “Short” cotton fiber elongation rates at different developmental stages. Error bars show the standard errors calculated from three replicates. * indicates a significant difference at *P = 0.01* between “Long” and “Short” at a given DPA

In this study, we measured the dynamic changes in FL of the two BILs at several fiber developmental time points (i.e., 5, 10, 13, and 19 DPA) to reveal the different features of FL development. The “Long” line had longer FL than the “Short” line and increased approximately exponentially from 5 to 19 DPA (Fig. 1a). The elongation rate at 10 DPA was shown to be the highest, with a significant difference in the FL between the two BILs (Fig. 1b). Therefore, 10 DPA developing fibers from these two BILs were chosen to study the molecular aspects of fiber elongation, although there may be other genes specifically expressed in fibers prior to or after 10 DPA.

### Overview of RNA sequencing data

To obtain a global insight into the characteristics of the transcriptome of elongating fibers, cDNA libraries for the fast elongation stage at 10 DPA were constructed and sequenced using an Illumina HiSeq 2000 platform. After trimming off the adapter sequences and removing the low-quality reads, we obtained 51,744,444 and 54,333,714 clean reads for “Long” and “Short”, respectively, with a single read length of 90 bp and a Q20 percentage (percentage of sequences with sequencing error rates lower than 1%) over 98% (Additional file 2: Table S2). The expressed genome in each line was represented by more than 4.65 Gb (billion bases), i.e., 58 times the size of the predicted transcript genome of the tetraploid genome (ca. 80 Mb). In total, 45,328,347 (87.6% of the clean reads) and 48,121,948 (88.6% of the clean reads) reads for “Long” and “Short”, respectively, were mapped to the TM-1 reference genome after alignment (Additional file 3: Table S3), resulting in the identification of 49,508 and 49,448 transcripts for the two BILs, respectively. This result suggests that 64.3% of the predicted genes (a total of 76,943 gene models in TM-1) are expressed in 10 DPA fibers.

### Differentially expressed genes with sequence variation

A total of 1551 DEGs (i.e., approximately 3% of the expressed genes in the two BILs) were identified between the “Long” and “Short” fiber transcriptomes at 10 DPA using the threshold FDR ≤ 0.001 and the absolute value of log2-fold change ≥1. These genes included 678 up-regulated and 873 down-regulated DEGs in “Long”, as compared to “Short”.

Because the sequence variation in coding sequences (CDSs) may change the sequence of the translated proteins and the function of the genes, we focused on the SNPs and insertions/deletions (InDels) that occurred in the CDSs. As a result, we identified 703 SNPs (about 1% of total number of genes predicted in Upland cotton) in 339 of the 1551 DEGs between the two BILs (Additional file 4: Table S4). Among these 339 DEGs with SNPs, 51.62% contained a single SNP, and 23.30%, 12.09%, 4.72%, and 8.27% DEGs contained 2, 3, 4, and more than 4 SNPs, respectively. These SNPs included 438 transitions (with 31.44% A/G and 30.87% C/T) and 265 transversions (with 8.39% A/C, 9.82% A/T, 9.96% G/C and 9.53% G/T). The SNPs were further divided into 256 synonymous SNPs in 179 genes and 447 non-synonymous SNPs in 251 genes. Among the non-synonymous SNPs, 435 SNPs were missense SNPs that change an amino acid into another, and 12 SNPs were nonsense SNPs that change an amino acid codon into a stop codon, resulting in a predicted truncated protein. Furthermore, 7 InDels were found in 7 DEGs (one each) between the two BILs (Additional file 5: Table S5).

**Fig. 2:**
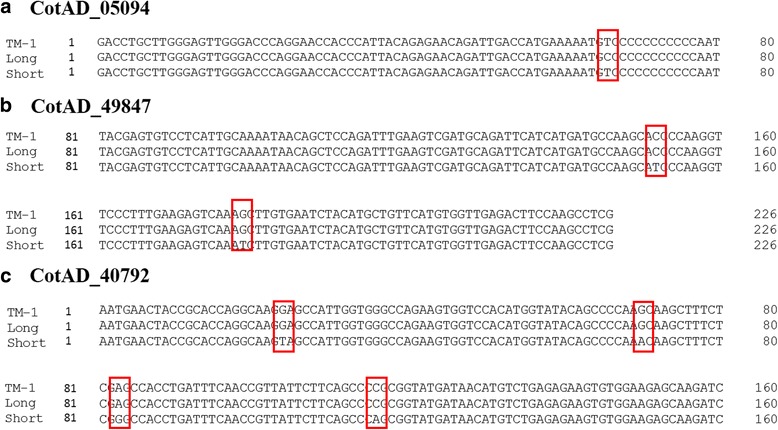
A comparison of sequences between genes from the TM-1 reference genome and the “Long” and “Short” genotypes from Sanger sequencing using PCR products. Vector sequences have been removed, only the sequences with SNP loci between the designed primers were retained. CotAD genes are from the CDS of the AD genome. **a** CotAD_05094; **b** CotAD_49847; **c** CotAD_40792

To validate the SNPs identified by the above analysis, we randomly chose three genes (CotAD_05094, CotAD_49847, and CotAD_40792) for PCR using gene specific primer pairs, cloning, and Sanger sequencing. The products of the selected SNP sites differed among “Long”, “Short” and the sequenced TM-1. One SNP site was predicted in the gene CotAD_05094 (with no function annotation), with a transition at the 66th base (T in TM-1 and “Short” → C in “Long”). This site was confirmed by the sequence analysis (Fig. 2a).

The predicted SNP site in CotAD_49847 (also coding for a functionally unknown protein LOC100788257) was a base transversion at the 179th position (G in TM-1 and “Long” → T in “Short”), which resulted in the non-synonymous replacement of lysine with asparagine. Another transition was present at the 152nd base (C in TM-1 and “Long” → T in “Short”), but this transition did not change the coded amino acid (Fig. 2b).

Four SNP sites were predicted in CotAD_40792 (coding for autophagy 9), including a transition at the 70th (G in TM-1 and “Long” → A in “Short”) and 83rd (A in TM-1 and “Long” → G in “Short”) bases and transversions at the 24th (G in TM-1 and “Long” → T in “Short”) and 116th (C in TM-1 and “Long” → A in “Short”) bases. All of these sites were confirmed by sequencing with no discrepancies. The 83rd and 116th bases had no changes in their coded amino acids; however, a change in the 24th base led to a stop codon, and the substitution at the 70th base changed serine to asparagine (Fig. 2c).

### DEGs with non-synonymous SNPs in FL QTL regions

To better understand the potential impact of DEGs with sequence variation, we focused on the 251 DEGs with 447 non-synonymous SNPs, while the 256 synonymous SNPs were not considered because of no changes in amino acids. A total of 257 SNP/InDels-containing DEGs were mapped onto the sequenced tetraploid cotton genome based on Zhang et al. [30]. A total of 109 DEGs were mapped to chromosome locations from A01 to A13 on the At subgenome; and 130 DEGs from D01 to D13 on the Dt subgenome. The remaining DEGs (i.e., 18 genes) were on unmapped scaffolds (Additional file 6: Figure S1). The numbers of DEGs with non-synonymous SNPs/InDels were distributed unevenly among 26 chromosomes, ranging from 2 to 24.

**Fig. 3:**
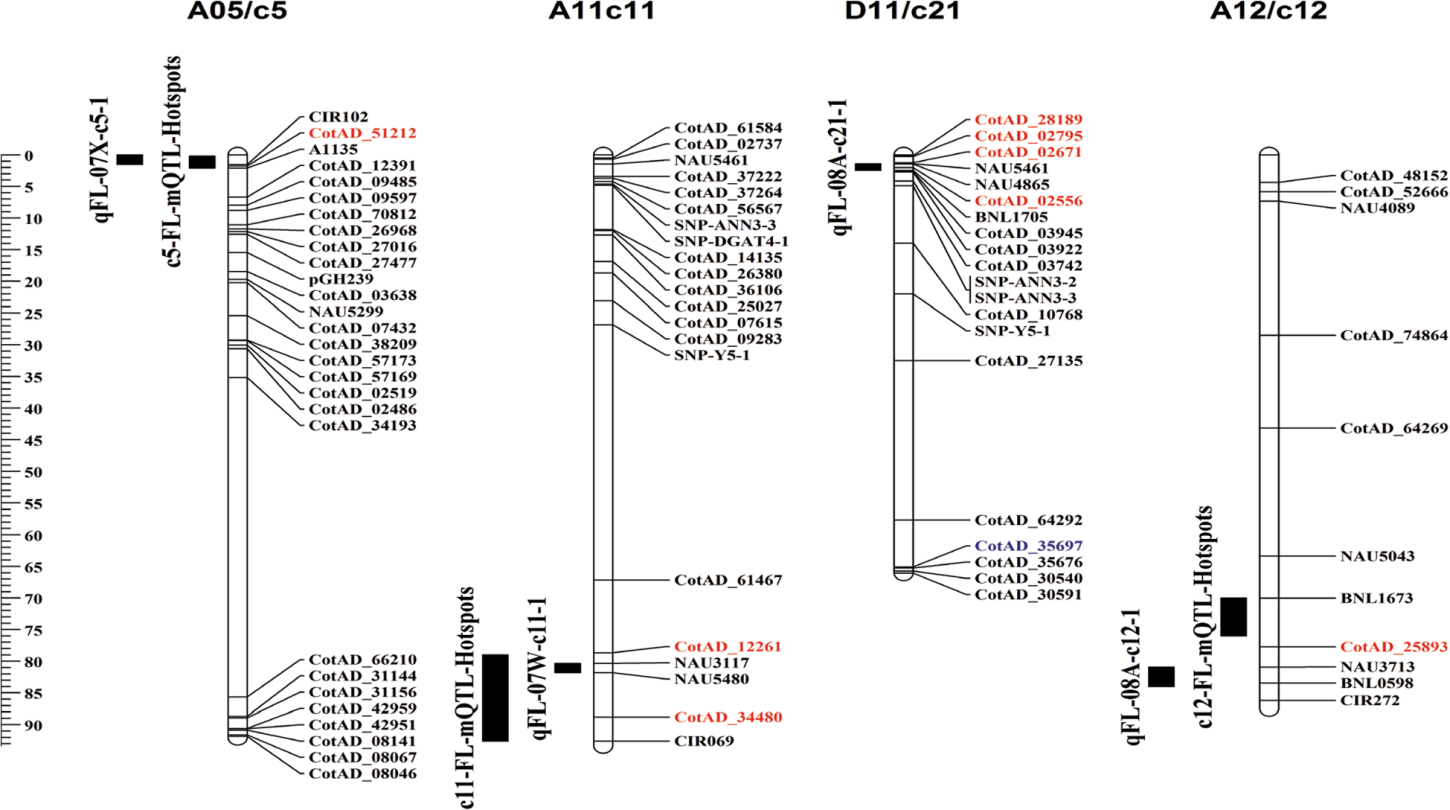
Comparative distributions of FL QTL hotspots or a QTL in cotton genome. c5-FL-mQTL-Hotspots refers to hotspots for FL QTL. qFL-07X-c5–1 refers to a FL QTL

Co-localization of QTL with DEGs will facilitate the identification of candidate genes for fiber quality traits such as FL. In our study, 8 DEGs with non-synonymous SNPs and no DEGs with InDels were mapped with the 4 FL QTLs that were previously reported in a BIL population [37] from which the “Long” and “Short” BILs were chosen, as shown in Table 1 and Fig. 3. The two BILs differed in SSR marker alleles in the four FL QTL regions, and the “Long” BIL carried the desirable QTL alleles for FL. The 4 FL QTL, i.e., qFL-07X-c5–1, qFL-07 W-c11–1, qFL-08A-c21–1, and qFL-08A-c12–1, were mapped onto A05, A11, D11, and A12, respectively (Table 1). The qFL-07X-c5–1 locus (on sequenced A05) had only 1 co-localized SNP-containing DEG encoding an ovate family protein 8 (CotAD_51212), and the SNP was an “T” in TM-1 and “Short” replaced by “C” in “Long”, indicating introgression of the gene from *G. barbadense* to Upland cotton. The qFL-07 W-c11–1 locus (on A11) had 2 co-localized DEGs encoding a putative aldo-keto reductase 1 (CotAD_12261) and a wave-dampened (*WVD2*)-like 1 protein (CotAD_34480). Similar to CotAD_51212, one of the two genes (CotAD_12261) had two SNPs between “Long” and “Short” (and TM-1), also indicating its *G. barbadense* origin, while one of the two SNPs in CotAD_34480 was from Upland cotton. The qFL-08A-c21–1 locus (on D11) had 4 co-localized DEGs encoding a proline -rich protein (PRP) (CotAD_02556), a D-cysteine desulfhydrase (CotAD_28189), a thaumatin-like protein (TLP) (CotAD_02795), and a CCT motif family protein (CotAD_02671). CotAD_02556 and CotAD_02671 had an apparent Upland cotton origin as the sequences from “Long” and TM-1 were the same. The qFL-08A-c12–1 (on A12) had 1 co-localized DEG encoding a flavin-binding monooxygenase family protein (CotAD_25893). The sequence variations of these 8 genes are shown in Additional file 7: Figure S2. As stated above, five of the 8 genes had a *G. barbadense* origin based on the identity of sequences between “Short” and TM-1, which were different from “Long”.

**Table 1:** A list of identified DEGs with non-synonymous SNPs that co-localized with 4 QTL for FL

Chromosome name	Co-localized QTL name	Co-localized gene name	FPKM		log2Ratio (L10/S10)	*P*-value	Putative gene function
			L10	S10			
A05/c5	qFL-07X-c5–1	CotAD_51212	0.60	3.51	-2.55	4.7E-12	transcription repressor OFP8-like [*Gossypium raimondii*]
A11/c11	qFL-07 W-c11–1	CotAD_12261	12.56	5.09	1.30	5.4E-21	putative aldo-keto reductase 1 [*Gossypium arboreum*]
		CotAD_34480	1.72	4.02	-1.22	9.1E-07	protein wvd2-like 1 [*Theobroma cacao*]
D11/c21	qFL-08A-c21–1	CotAD_02556	29.97	74.82	-1.32	1.4E-43	proline-rich protein [*Gossypium hirsutum*]
		CotAD_28189	1.73	10.56	-2.61	4.4E-40	putative D-cysteine desulfhydrase 1[*Gossypium raimondii*]
		CotAD_02795	105.17	248.10	-1.24	4.7E-222	thaumatin-like protein [*Gossypium raimondii*]
		CotAD_02671	13.82	27.87	-1.01	9.2E-23	CCT motif family protein [*Theobroma cacao*]
A12/c12	qFL-08A-c12–1	CotAD_25893	16.47	6.31	1.39	6.3E-35	flavin-containing monooxygenase family protein [*Theobroma cacao*]

The BIL population that included the two BILs used in this study only mapped 4 QTL for FL, each of which explained 8.23–16.72% of the phenotypic variation [37]. Interestingly, after their chromosomal locations were compared with the 13 FL QTL hotspots (each with 4 or more FL QTL identified) from an updated Cotton QTLdb database (Release 2.0) [36], 3 of the 4 FL QTL mapped in the BIL population were also located in three FL QTL hotspot regions. These three FL QTL hotspots, each with 4 QTL, were as follows: c5-FL-mQTL-hotspot on A05 (containing qFL-07X-c5–1), c11-FL-mQTL-hotspot on A11 (containing qFL-07 W-c11–1), and c12-FL-mQTL-hotspot on A12 (containing qFL-08A-c12–1). No FL QTL hotspot was found to encompass the QTL qFL-08A-c21–1 locus on D11. These results indicated the reliability of the QTL mapped in the BIL population and provided high confidence in the current study.

### Analysis of co-localized SNP-containing DEGs for FL QTL

**Fig. 4:**
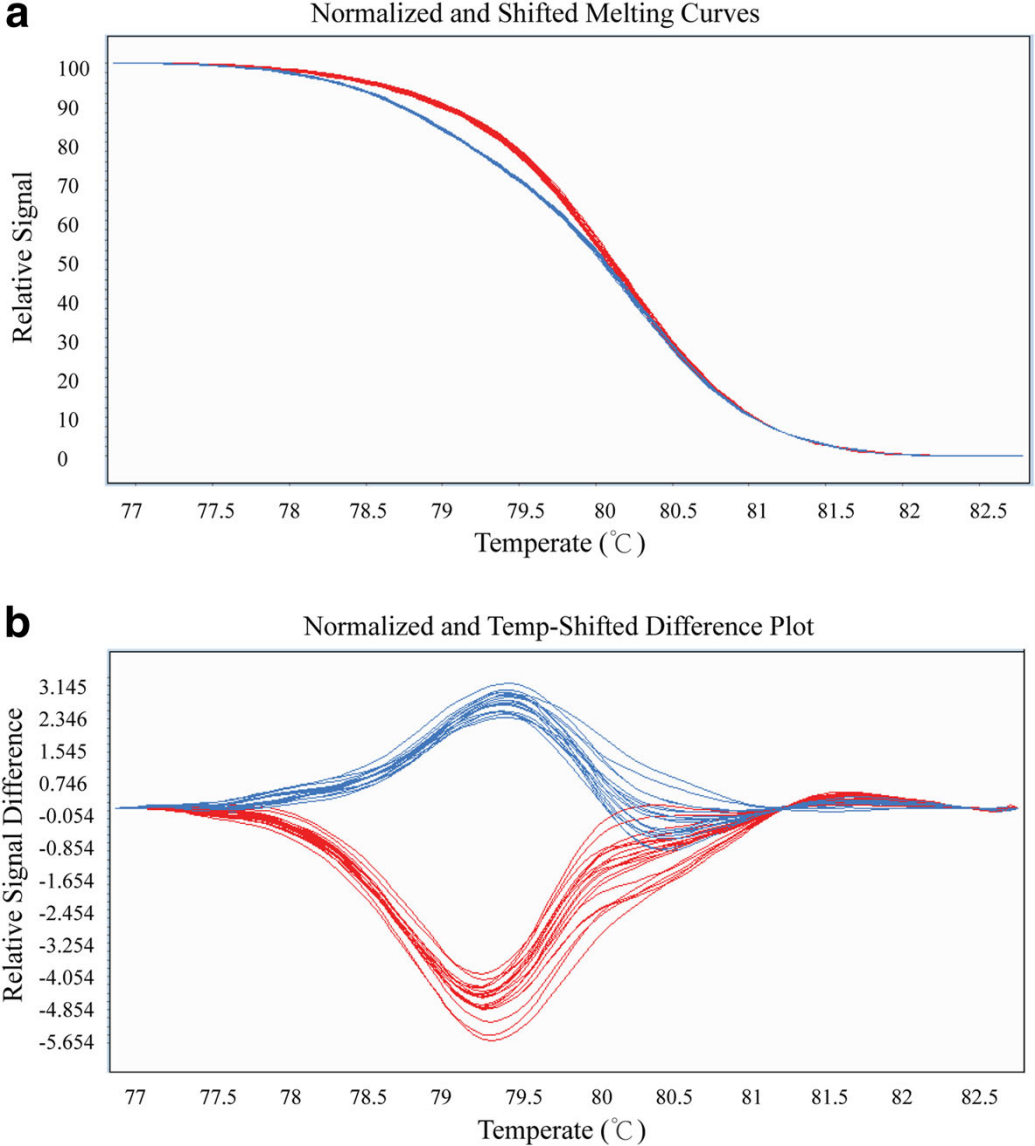
A HRM analysis to confirm the presence of single nucleotide polymorphisms of CotAD_28189 at position 879 nt in a subset of a backcross inbred line population. **a** Original melting curves. **b** Melting curves after logarithm calculations. Blue and red curves correspond to “Long”, “Short” genotypes, respectively

To validate whether DEGs with SNPs had any associations with FL measured in 6 environments from 2006 to 2009 [37], we designed 16 primers (Additional file 8: Table S6) for the non-synonymous SNPs for these 8 FL-QTL co-localized DEGs. Then, a single strand conformation polymorphism (SSCP) and a high-resolution melting (HRM) analysis were used to genotype the 146 BILs from which the two BILs were selected. While the analyses did not detect any polymorphisms in 5 DEGs within the BIL population, 4 SNPs in 3 other DEGs (i.e., 1 SNP in CotAD_02556, 2 SNPs in CotAD_02795, and 3 SNPs in CotAD_28189) that were co-localized with the qFL-08A-c21–1 locus (on D11) significantly correlated with FL tested in one or more environments (Table 2). Specifically, the SNP at the 474 nucleotide (nt) position with an A to G replacement (leading to lysine to arginine change) on CotAD_02795 highly significantly (at *P = 0.01*) correlated with FL tested in all of the six testing environments (i.e., in Anyang from 2006 to 2009 and in Wangjiang and Xinjiang in 2007). Therefore, this SNP conferred a consistent effect on FL across different environments, indicating a high likelihood of the gene CotAD_02795 as a candidate gene for FL QTL qFL-08A-c21–1. This QTL was located on chromosome c21, i.e., D11. An example of an HRM assay of the SNP at 879 nt of CotAD_28189 in a subset of the 146 BILs is shown in Fig. 4.

**Table 2:** Non-synonymous SNP markers in three genes correlated with fiber Length

Gene Name	SNP position based on CDS sequence	Variant	Amino acid	Trait and Environment^a^	QTL name
CotAD_02556	241	A/C	Lys- > Gln	FL-08-A (0.180*)	qFL-08A-c21–1
CotAD_28189	147	C/G	Asp- > Glu	No correlation with FL	qFL-08A-c21–1
	336	C/A	Asp- > Glu	No correlation with FL	qFL-08A-c21–1
	879	T/C	Tyr- > His	FL-07-A (0.189*)	qFL-08A-c21–1
				FL-08-A (0.179*)	
CotAD_02795	156	A/G	Asn- > Ser	FL-09-A (0.211*)	qFL-08A-c21–1
	474	A/G	Lys- > Arg	FL-06-A (0.216**)	qFL-08A-c21–1
				FL-07-A (0.281**)	
				FL-07-X (0.277**)	
				FL-07-W (0.277**)	
				FL-08-A (0.248*)	
				FL-09-A(0.311**)	

Correction coefficients between trait and SNP loci was tested by SPSS software (*, *P* < *0.05*, **, *P* < *0.01*).

^a^ FL, fiber length; 06, 2006; 07, 2007; 08, 2008; 09, 2009; A, Anyang; X, Xinjiang; W, Wangjiang

We further used a quantitative RT-PCR (qRT-PCR) analysis to validate the three DEGs identified by RNA-Seq with primers list in Additional file 8: Table S6. The results showed that the 3 DEGs had significant differential expression between the two BILs at the 0.05 level, which was in accordance with the RNA-Seq expression results at 10 DPA.

**Fig. 5:**
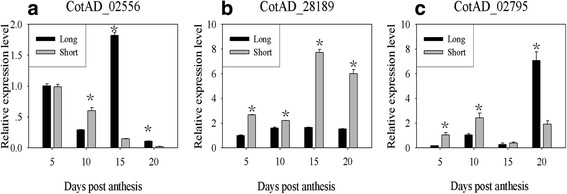
qRT-PCR expression levels of 3 FL candidate genes. **a**-**c** Expression leve﻿ls of gene CotAD_02556, CotAD_28189, and CotAD_02795, respectively. The x-axis represents developmental stages (5, 10, 15, and 20 DPA), and the y-axis indicates the relative expression levels as determined by qRT-PCR. The error bars shown are the means of three biological replicates. * indicates a significant difference at *P = 0.01* between “Long” and “Short” at a given DPA

The differential expression modes of the three genes during different fiber elongation stages (i.e., 5, 10, 15, and 20 DPA) were further analyzed (Fig. 5). CotAD_02556 (encoding for PRP) had decreased expression levels in fibers from 5 to 20 DPA in both “Long” and “Short”, except for “Long” at 15 DPA when it had the highest level of expression. The two lines had similar level of gene expression at 5 DPA; however, “Long” had a significantly lower level of expression at 10 DPA, followed by significantly reduced expression at 15 and 20 (Fig. 5a). CotAD_28189 (encoding for a putative D-cysteine desulfhydrase 1) and CotAD_02795 (encoding for TLP) had lower transcript levels in both lines at 5 and 10 DPA, but much higher expression levels at 15 and 20 DPA. Compared to “Short”, both genes were down-regulated in “Long” during the 4 fiber development stages (Fig. 5b and c), except at 20 DPA when CotAD_02795 was up-regulated in “Long”. Because *G. barbadense* has a longer fiber elongation period than *G. hirsutum* [10], the up-regulation of this gene at 20 DPA or a late stage may be related to the longer fibers of *G. barbadense* and “Long” in this current study.

## Discussion

### The fiber transcriptome in “long” and “short” BILs with the tetraploid cotton genome sequence as a reference

We used RNA-Seq to investigate the transcriptome profiling of two BILs using the published tetraploid genome sequence as the reference for annotating expressed genes [29, 30]. More than 87% of the clean reads for the “Long” and “Short” BILs were mapped to the reference genome, which is much higher than the average of 70% reads uniquely mapped to the *G. raimondii* genome reported previously [20]. Furthermore, 64.3% of the 76,943 gene models in the sequenced TM-1 genome were expressed in 10 DPA fibers, consistent with previous reports [26]. The present results provide a most comprehensive overview of gene expression at the transcriptomic level during the fastest fiber development stage.

In this study, we chose 10 DPA fibers to investigate fiber elongation with two BILs that differ only in FL and its related micronaire (i.e., fiber fineness) trait. As reported previously, cotton fiber elongation lasts up to 25 DPA, while FM is predominantly determined during the secondary cell wall synthesis stage at 15–35 DPA [38]. Therefore, genes identified at 10 DPA would reflect these that are likely related to fiber elongation, which was the focus of this current study. It should be recognized that with more time-points during fiber development from fiber initiation to maturity from a set of BILs or NILs differing in FL or other fiber quality traits, a comprehensive transcriptome atlas can be investigated. However, through a transcriptome analysis and DEG and SNP identification, in combination with physical mapping and co-localization of FL-QTL and DEGs with SNPs, several candidate genes especially CotAD_02795 possibly underlying the genetic control of FL differences between *G. barbadense* and *G. hirsutum* have been identified in this study. This study has paved the way to search for candidate genes for fiber traits in cotton in the future.

### A genome-wide coding sequence-based SNP discovery based on RNA-Seq

In the current study, 1551 DEGs between the “Long” and “Short” BILs in the 10 DPA fiber transcriptome were identified, and 339 of these DEGs contained 703 SNPs (including 256 synonymous and 447 non-synonymous with 12 nonsense and 435 missense). The proportion of DEGs with SNPs was consistent with marker polymorphism results based on more than 2300 SSR and SNP markers. These DEGs with SNPs were further mapped to the sequenced tetraploid cotton TM-1 genome [30]. Most importantly, 8 genes with SNPs were co-localized with 4 FL QTL (3 of which were in the QTL hotspot regions for FL) identified in a BIL population from which the two BILs were chosen. The mining of DEGs with SNPs in these QTL and QTL hotspot regions helped to identify candidate genes for FL. This positional candidate gene approach based on QTL mapping and sequenced genome physical mapping represents one of the most efficient strategies to narrow the number of candidate genes for a trait of interest.

### Putative functions of candidate genes for FL

After correlation and qRT-PCR analyses, 3 candidate genes for a FL QTL on chromosome c21 (D11) were identified in cotton in this study. As the cell wall structure protein, the proline-rich protein (PRP) participates in plant growth and development [39]. In cotton, several PRPs were previously isolated, but none was functionally characterized except for GhPRP5 [9, 40]. The GhPRP5 protein, as a negative regulator, participates in modulating cotton fiber elongation [41] and interacts with auxin-responsive family protein. Auxin plays an important role in fiber initiation; the number of lint fibers was increased when the *iaaM* gene was overexpressed [42]. In our study, a non-synonymous SNP was found in CotAD_02556 (PRP), which correlated with FL tested in only one of the six environments. Since its expression was down-regulated in “Long” at 10 DPA compared to “Short”, and its expression level was lower at 10 DPA than at 5 DPA, this PRP gene may also function as a negative regulator of FL in natural variation.

Another gene, CotAD_28189, encoding a D-cysteine desulfhydrase, catalyzes D-cysteine into pyruvate. Pyruvate is a key intermediate of glucose, fatty acids and amino acid metabolism. Pyruvate is also related to VLCFA biosynthesis and phenylpropanoid metabolism, and VLCFA promotes cotton fiber and *Arabidopsis* cell length via activating ethylene biosynthesis [43]. This gene showed increased relative expression levels from 0 to 25 DPA and was down-regulated in “Long” compared with “Short”. A correlation analysis also showed that, of the three non-synonymous SNPs identified in this gene, one SNP at 879 nt (T to C replacement and tyrosine to histidine change) was correlated with FL tested in only two of the six environments. Thus, similar to CotAD_02556, gene encoding for D-cysteine desulfhydrase may be a important candidate gene for FL.

The most important candidate gene for FL is CotAD_02795 encoding for a thaumatin-like protein (TLP). Plant TLPs are a family of pathogenesis-related genes. Tu et al. [44] proposed that two *GbTLPs* participate in secondary cell wall synthesis, and then Munis et al. [45] confirmed this hypothesis. A previous research suggested that *TLP3* may be a candidate gene for superior FL and FS [46]. In this study, we found that, even though both non-synonymous SNPs at 156 nt and 474 nt in this gene (CotAD_02795) had significantly correlated with FL, the most consistent effect on FL across all the testing environments was conferred by the SNP at 474 nt for the A to G (lysine to arginine change) replacement. Further studies are needed to reveal the role of this gene with regard to the natural variation in FL.

## Conclusions

Based on the comparative RNA-Seq analysis of developing fibers between two BILs differing in FL during the fast elongation period, with the AD1 genome as a reference, 678 genes were up-regulated and 873 genes were down-regulated in the “Long” line. According to the FL QTL and hotspots reported previously, we identified 8 genes with SNP loci in CDS regions. Proline-rich protein, thaumatin-like protein, and D-cysteine desulfhydrase are potential candidate genes responsible for a FL QTL on chromosome 21. These findings may serve as the foundation for further in-depth studies of the molecular mechanism of natural variation in fiber elongation.

## Methods

### Plant materials and growth conditions

Two BIL lines, NMGA-062 and NMGA-105, named “Long” and “Short”, respectively, were planted at the Institute of Cotton Research, Chinese Academy of Agricultural Science, Anyang, Henan Province, China. The two lines were included in a panel of 146 BILs arranged with three replications and single row plots and tested in five environments (Anyang in 2006, 2007, 2008 and 2009, Wangjiang and Xinjiang in 2007) as described previously [37]. Briefly, the BILs were developed from cross between *G. hirsutum* SG747 as the recurrent parent and *G. barbadense* Giza75 followed by 2 generations of backcrosses and then 4 times of self-pollination in New Mexico State University, Las Cruces, NM, USA. Mature fiber samples from each year were harvested from at least 25 plants (1 boll per plant, from the middle branch of the plant) of each line, and fiber quality traits were evaluated using a high volume instrument (HVI) 900 at the Test Center of Cotton Fiber Quality affiliated with the Agriculture Ministry of China, Institute of Cotton Research, Chinese Academy of Agricultural Science, Anyang, Henan, China. Fibers at 5, 10, 13 and 19 DPA were measured using a previously published method [10].

### RNA isolation and RNA-Seq

Flowers were tagged on the day of anthesis from the field-grown plants. Fibers were collected from 5, 10, 15, 20 DPA ovules and dissected. The excised ovules and fiber were frozen in liquid nitrogen and stored at −80 °C until used for total RNA extraction. RNA was individually isolated from different developmental stage fibers of 15 bolls in “Long” and “Short” (each with three biological replications) using an RNAprep Pure Plant Kit (Tiangen, China). RNA quality and quantity were confirmed using an Agilent 2100 Bioanalyzer. Extracted RNA samples were selected based on an RNA integrity number (RIN) higher than 8, spectroscopic A260/A280 nm readings between 1.9 and 2.1, and A260/A230 nm readings larger than 1.8.

cDNA libraries representing the elongation (10 DPA) periods of the two BILs, NMGA-062 (“Long”) and NMGA-105 (“Short”), were constructed and sequenced using an Illumina HiSeq 2000 platform as described by Ma et al. [47]. All the short reads were deposited in the National Center for Biotechnology Information (NCBI) database and can be accessed in the Short Read Archive (SRA) (accession number SRP 039385).

### Data processing and mapping reads to the genome

We obtained clean reads by removing the adapter reads and the low-quality reads, which included the reads with more than 5% unknown nucleotides and reads with more than 20% nucleotides with sequencing quality ≤10.

The assembled cotton (TM-1, the genetic standard line for Upland cotton) genome sequence [29] was used as a reference for paired-end reads mapping. SOAP2 [48] software was used to align the filtered clean reads to genes of the genome with default parameters.

### Differential gene expression analysis

The expression level of each gene was calculated as fragments per kilobase transcriptome per million mapped reads (FPKM) by normalizing for the length of the gene and for the number of mapped reads [49]. The significance of DEGs between the “Long” and “Short” BILs was judged using the threshold FDR ≤ 0.001 and the absolute value of log2-fold change ≥1 using Cuffdiff v2.1.1 [50].

### In-silico SNP discovery and validation of predicted SNPs using single-strand conformation polymorphism (SSCP) analysis and Sanger sequencing

In-silico SNP detection was performed by mapping paired-end reads of the two BILs back to the TM-1 genome using SOAPsnp [51] with read depth ≥ 10 and mapping quality ≥20. Only the unique matched reads were used to identify homolog SNPs.

For SNP validation, genomic DNA was extracted from seedlings of the “Long” and “Short” BILs used in RNA-Seq and from seedlings of TM-1. We used Primer Premier 6 to design primers with the following criteria: primer length between 18 to 22 bp; GC content between 40% to 60%; Tm temperature at approximately 58 °C, and PCR product size between 100 to 250 bp. PCR amplification and detection of polymorphisms between the “Long” and “Short” BILs were performed with the SSCP technology previously described [34]. The purified PCR products were linked to the pMD18-T vector (TaKaRa, Japan) and sequenced from both ends using M13F and M13R primers with more than six clones per PCR product. Then, ClustalX2 software was used to compare the sequences obtained by Sanger sequencing.

### Chromosome location of DEGs with non-synonymous SNPs in FL QTL and QTL hotspots through linkage mapping and physical mapping

In our previous study [37], 4 FL QTLs were mapped using SSR markers in a BIL population from which the “Long” and “Short” BILs were chosen. To physically locate these 4 QTLs and other fiber length QTLs, the SSR marker sequence data were downloaded from the CottonGen database (https://www.cottongen.org/data/download/marker) [52]. We collected the marker names in the published linkage map of [“Guazuncho2” (*G. hirsutum*) × “VH8–4602” (*G. barbadense*)] in a reported meta-analysis of QTL [36]. Then, the chromosome locations of the markers and DEGs were identified using the BLAST program with the marker and DEG sequences as the query and the genome sequence by Zhang et al. [30] as the subject. The positions of the 4 FL QTL regions and the corresponding hotspots for FL in chromosomes were determined using the anchoring marker’s location in the map. DEGs with non-synonymous SNPs in FL QTL and QTL hotspots were selected for further study.

### HRM analysis of SNP markers and correlation with FL

The BIL population of 146 lines from which the two sequenced BILs were selected was used for a HRM analysis [47]. Each SNP primer was designed based on the sequence of DEGs. The 20-μL HRM reaction mixture was performed on the LightCycler480 with LightCycler^®^ 480 High Resolution Melting Master (Roche, Germany). LightCycler 480 Gene Scanning software was used to genotype all the samples. The polymorphic markers were coded:) “0” for absence and “1” for presence and used for correlation analysis (using SPSS) with FL in the BIL population.

### Quantitative RT-PCR (qRT-PCR) analysis

Total RNA was extracted from the 4 fiber development stages (i.e., 5, 10, 15, and 20 DPA) of cotton fibers. Gene-specific primers were designed using Primer Premier 6. The first-strand cDNA was synthesized using a PrimeScript® RT Reagent Kit (Perfect Real Time) (TaKaRa, Japan) according to the manufacturer’s protocol. The cDNA templates were diluted 8-fold and used for qRT-PCR. The qRT-PCR experiment with three technical replicates was performed on a Mastercycler® ep realplex (Eppendorf, Germany). The PCR program was as follows: an initial denaturation step of 10 min at 95 °C, followed by 40 cycles of 15 s at 95 °C for denaturation, 25 s at 60 °C for annealing and 30 s at 72 °C for extension. Then, melting curve analysis was performed. The relative expression levels of candidate genes were measured using the 2-^△△CT^ method and normalized to the histone-3 gene (AF024716).

## Additional files

Additional file 1: Table S1. Average fiber quality of the two BILs tested in different environments based on Yu et al. [37]. (DOC 15 kb)

Additional file 2: Table S2. Summary of RNA-Seq data. (DOC 30 kb)

Additional file 3: Table S3. Summary of RNA-Seq mapping reads to the TM-1 reference genome. (DOC 38 kb)

Additional file 4: Table S4. Single nucleotide polymorphism (SNP) site overview. A list of 703 SNP sites identified between “Long” and “Short” in 339 genes. (XLS 172 kb)

Additional file 5: Table S5. InDels (Insertions/deletions) between “Long” and “Short” identified in this study. (XLS 27 kb)

Additional file 6: Figure S1. Chromosome distribution of 239 DEGs with SNP/InDels between “Long” and “Short” in the *Gossypium hirsutum* genome from A01 to A13, and from D01 to D13. Genes with InDels are indicated in blue. (PDF 1348 kb)

Additional file 7: Figure S2. Comparison of 8 co-localized gene sequences obtained by TM-1 genome sequencing and SNP loci that exist in the “Long” and “Short” genotypes. (BMP 24574 kb)

Additional file 8: Table S6. A list of primers used for SSCP, HRM and qRT-PCR analyses. (DOC 43 kb)

## Abbreviations

BILs: Backcross inbred lines
CSILs: Chromosome segment introgression lines
DEGs: Differentially expressed genes
DPA: Days post anthesis
FL: Fiber length
FM: Fiber micronaire
HRM: High-resolution melting
InDels: Insertions/deletions
NILs: Near-isogenic lines
QTL: Quantitative trait loci
SNP: Single nucleotide polymorphism
SSCP: Single strand conformation polymorphism
