# Supplementary material for: Identification of candidate genes for fiber length quantitative trait loci through RNA-Seq and linkage and physical mapping in cotton
Source: BMC Genomics. 2017 May 31;18:427. doi: 10.1186/s12864-017-3812-5 (PMC5452627; doi:10.1186/s12864-017-3812-5)
Supplement: Supplementary file 1 — Average fiber quality of the two BILs tested in different environments based on Yu et al. [37]. (DOC 15 kb) [file 12864_2017_3812_MOESM1_ESM.doc]

| **Additional file 1: Table S1. Average fiber quality of the two BILs tested in different environments based on Yu et al [37]** | | | | | |  |  |  |
| --- | --- | --- | --- | --- | --- | --- | --- | --- |
|  | | Fiber  length  (mm) | Fiber  strength  (cN/tex) | Micronaire  (unit) | Fiber  elongation  (%) | Fiber uniformity  (%) | | |
| “Long” | | 32.09±0.42** | 32.15±0.72 | 4.19±0.17** | 6.53±0.24 | 83.62±0.72 | | |
| “Short” | | 27.21±0.71 | 31.11±0.49 | 5.02±0.08 | 6.32±0.40 | 83.57±0.29 | | |

**Long line has significant difference with Short line at the 1% level.
