# Supplementary material for: Identification of candidate genes for fiber length quantitative trait loci through RNA-Seq and linkage and physical mapping in cotton
Source: BMC Genomics. 2017 May 31;18:427. doi: 10.1186/s12864-017-3812-5 (PMC5452627; doi:10.1186/s12864-017-3812-5)
Supplement: Supplementary file 2 — Summary of RNA-Seq data. (DOC 30 kb) [file 12864_2017_3812_MOESM2_ESM.doc]

| **Additional file 2: Table S2. Summary of RNA-Seq data** | | | | | | | |
| --- | --- | --- | --- | --- | --- | --- | --- |
| Sample | Raw reads | Total clean reads | Total nucleotides (nt) | Q20 % | N % | GC % |  |
| "Long" | 54,839,126 | 51,744,444 | 4,656,999,960 | 98.84 | 0.00 | 47.55 |  |
| "Short" | 57,816,334 | 54,333,714 | 4,890,034,260 | 98.55 | 0.00 | 48.36 |  |
