# Supplementary material for: Identification of candidate genes for fiber length quantitative trait loci through RNA-Seq and linkage and physical mapping in cotton
Source: BMC Genomics. 2017 May 31;18:427. doi: 10.1186/s12864-017-3812-5 (PMC5452627; doi:10.1186/s12864-017-3812-5)
Supplement: Supplementary file 3 — Summary of RNA-Seq mapping reads to the TM-1 reference genome. (DOC 38 kb) [file 12864_2017_3812_MOESM3_ESM.doc]

| **Additional file 3: Table S3.** **Summary of RNA-Seq mapping reads to the TM-1 reference genome** | | | | | | | | | | | |
| --- | --- | --- | --- | --- | --- | --- | --- | --- | --- | --- | --- |
|  | Map to the TM-1 genome | | | | |  | Map to the TM-1 gene | | | | |
|  | "Long" | |  | "Short" | |  | "Long" | |  | "Short" | |
|  | Reads number | Percentage |  | Reads number | Percentage |  | Reads number | Percentage |  | Reads number | Percentage |
| Total reads | 51744444 | 100.00% |  | 54333714 | 100.00% |  | 51744444 | 100.00% |  | 54333714 | 100.00% |
| Total base pairs | 4656999960 | 100.00% |  | 4890034260 | 100.00% |  | 4656999960 | 100.00% |  | 4890034260 | 100.00% |
| Total mapped reads | 45328347 | 87.60% |  | 48121948 | 88.57% |  | 32560548 | 62.93% |  | 33705880 | 62.03% |
| Perfect match | 35527567 | 68.66% |  | 37468247 | 68.96% |  | 24123087 | 46.62% |  | 24289201 | 44.70% |
| <=5bp mismatch | 9800780 | 18.94% |  | 10653701 | 19.61% |  | 8437461 | 16.31% |  | 9416679 | 17.33% |
| Unique match | 34323773 | 66.33% |  | 32706181 | 60.20% |  | 24122814 | 46.62% |  | 23193958 | 42.69% |
| Multi-position match | 11004574 | 21.27% |  | 15415767 | 28.37% |  | 8437734 | 16.31% |  | 10511922 | 19.35% |
| Total unmapped reads | 6416097 | 12.40% |  | 6211766 | 11.43% |  | 19183896 | 37.07% |  | 20627834 | 37.97% |
