# Supplementary figures and images for: Identification of candidate genes for fiber length quantitative trait loci through RNA-Seq and linkage and physical mapping in cotton
Source: BMC Genomics. 2017 May 31;18:427. doi: 10.1186/s12864-017-3812-5 (PMC5452627; doi:10.1186/s12864-017-3812-5)

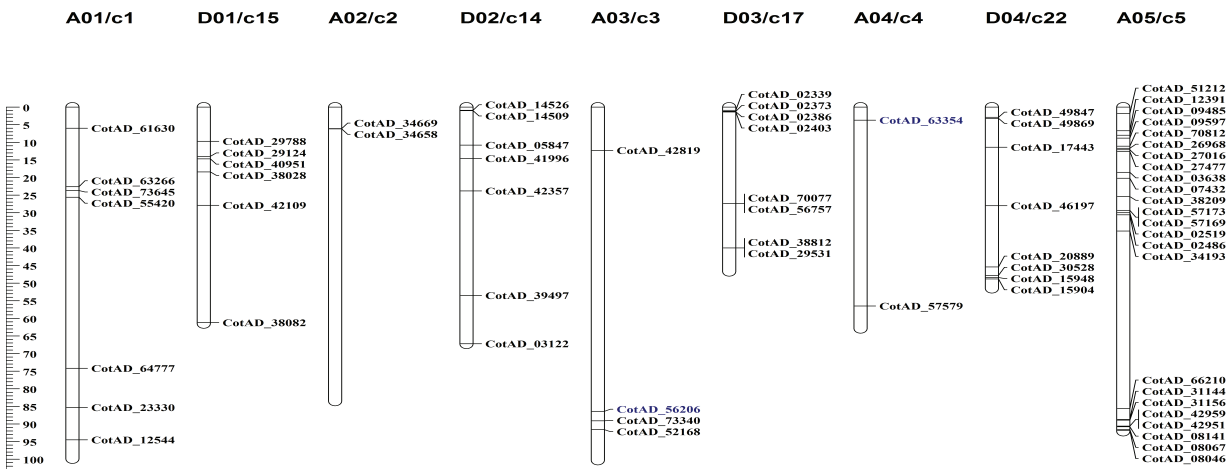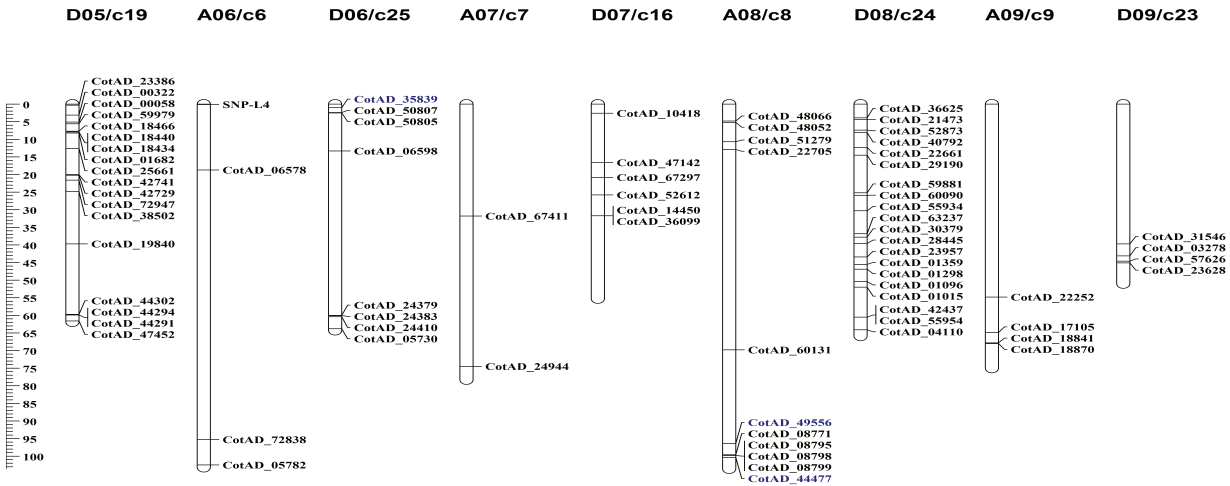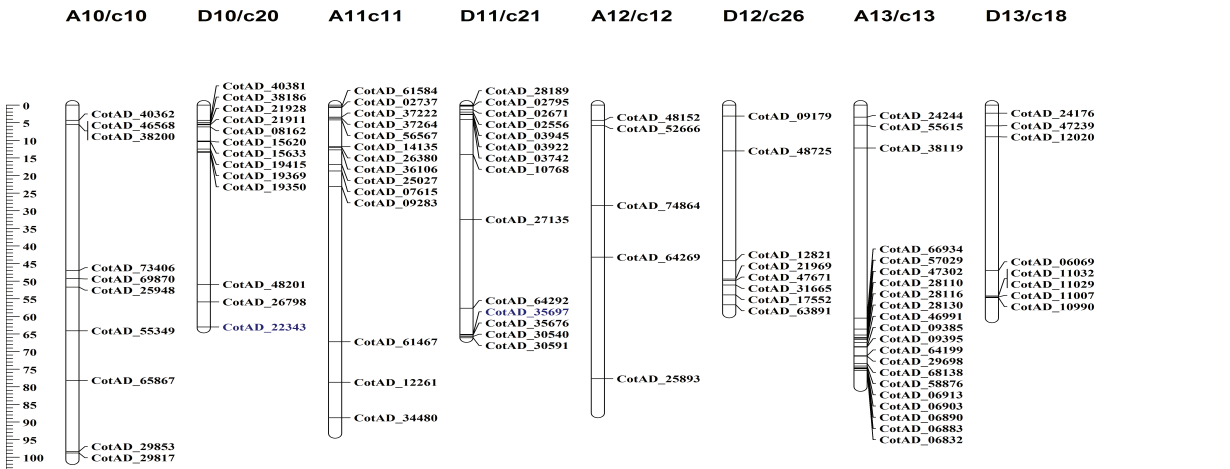

Supplement: Supplementary file 6 — Chromosome distribution of 239 DEGs with SNP/InDels between “Long” and “Short” in the Gossypium hirsutum genome from A01 to A13, and from D01 to D13. Genes with InDels are indicated in blue. (PDF 1348 kb) [file 12864_2017_3812_MOESM6_ESM.pdf]

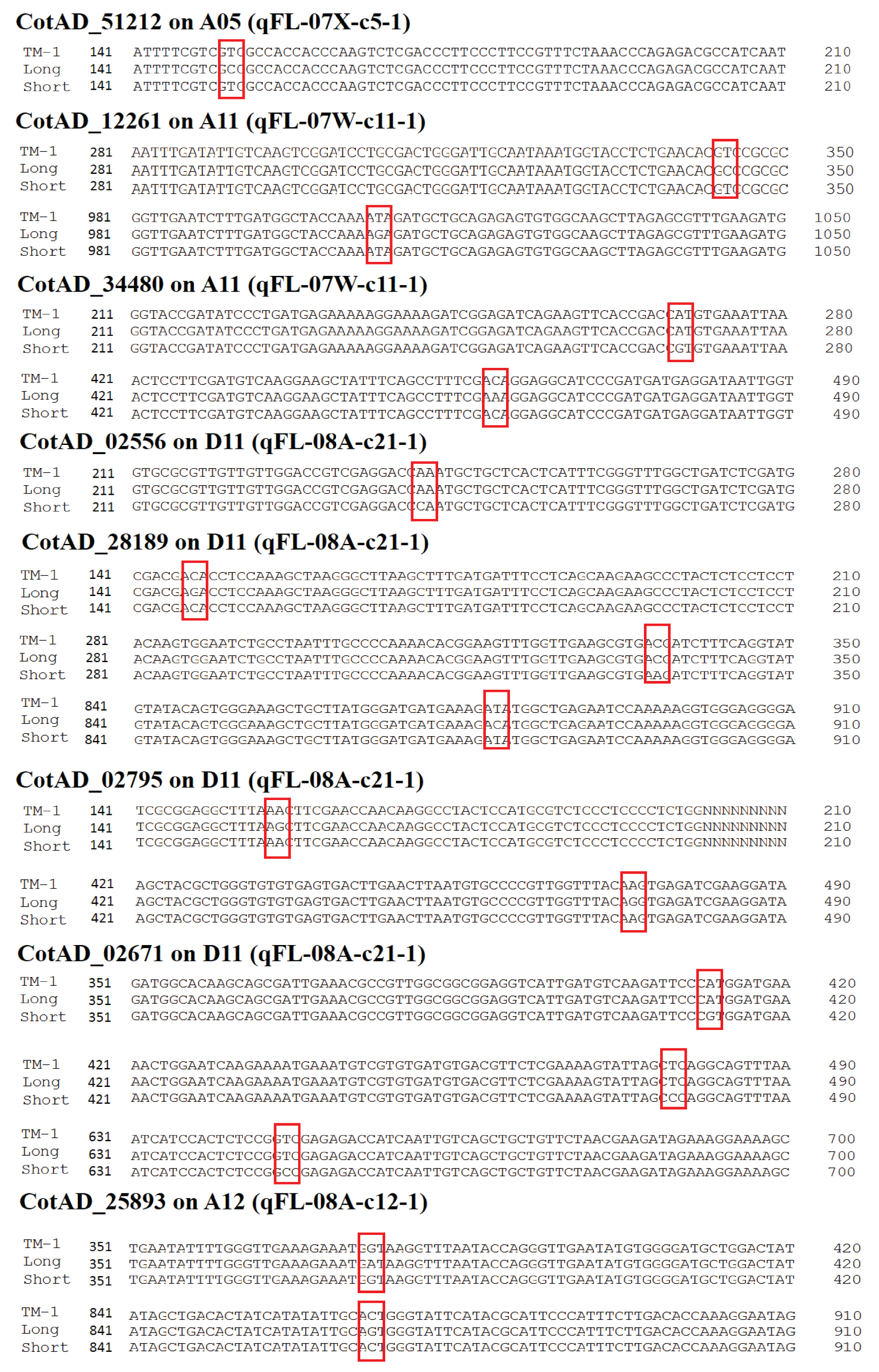

Supplement: Supplementary file 7 — Comparison of 8 co-localized gene sequences obtained by TM-1 genome sequencing and SNP loci that exist in the “Long” and “Short” genotypes. (BMP 24574 kb) [file 12864_2017_3812_MOESM7_ESM.bmp]
