# Supplementary material for: Identification of candidate genes for fiber length quantitative trait loci through RNA-Seq and linkage and physical mapping in cotton
Source: BMC Genomics. 2017 May 31;18:427. doi: 10.1186/s12864-017-3812-5 (PMC5452627; doi:10.1186/s12864-017-3812-5)
Supplement: Supplementary file 8 — A list of primers used for SSCP, HRM and qRT-PCR analyses. (DOC 43 kb) [file 12864_2017_3812_MOESM8_ESM.doc]

| **Gene** | **Forward primers (5’ to 3’)** | **Reverse primers (5’ to 3’)** |
| --- | --- | --- |
| **SSCP analysis** |  |  |
| CotAD_05094 | GACCTGCTTGGGAGTTGGGA | GGTGGATGAGGCTTGGGACT |
| CotAD_49847 | ACGCCGCCACAACCAATAGA | ACGAGGCTTGGAAGTCTCAACC |
| CotAD_40792 | AATGAACTACCGCACCAGGCA | CCTCTGAGAATCCCTCCAGTCA |
| **HRM analysis** |  |  |
| CotAD_02556-241 | GTTGTTGTTGGACCGTCGAGGA | GCAAAGGCAAACAGCAGCATCG |
| CotAD_02671-412 | TTGGCGGCGGAGGTCATTGA | TCGAGAACGTCACATCACACGA |
| CotAD_02671-478 | GTCGTGTGATGTGACGTTCTCG | AGCAGCCTGTTGCATCCATTCC |
| CotAD_02671-647 | ATGACAGACTCTCGGCAACGG | AGCTGACAATTGATGGTCTCTC |
| CotAD_02795-156 | CAAGTGCCCTCACCCAGTGT | AGGGAGACGCATGGAGTAGG |
| CotAD_02795-474 | CGCTGGGTGTGTGAGTGACTTG | GCCACCACTCGCTTGTTATCCT |
| CotAD_12261-344 | ATTGTCAAGTCGGATCCTGCGA | ACATCAAGGCGCTTCAGGCTAG |
| CotAD_12261-1007 | GTGACGGTTGAATCTTTGATGGC | CTTCAAACGCTCTAAGCTTGCCA |
| CotAD_25893-376 | CACAAGGAGCTGTGGTTGTATCT | AAACACCATAGTCCAGCATCCC |
| CotAD_25893-866 | GGCTCTTGTGTCATAGCTGAC | GGGTGCTTAGAAGACGATTTCT |
| CotAD_28189-147 | AACCAACGACGACACCTCCAA | GAGGAGAGTAGGGCTTCTTGCT |
| CotAD_28189-336 | TGCTCTTTGGTACTCACGATCT | TGCTTCCGCCAATAAGAACTCC |
| CotAD_28189-879 | AGTGGGAAAGCTGCTTATGGGA | AACCCAAGGAGTCCACCTGTGT |
| CotAD_34480-269 | GGAGATCAGAAGTTCACCGACC | CGAATATCTCCGGCACCACCAG |
| CotAD_34480-459 | GCGAAGCTCTCCTCTGACTCCT | CCTCATCATCGGGATGCCTCCT |
| CotAD_51212-151 | ACGGCTTCCATTTGGTCGAACC | GGAGCATTGATGGCGTCTCTGG |
| **qRT-PCR analysis** | |  |
| CotAD_02556 | CGCTGCTCTTCTTCGTACCT | GCTCTTGTGGGTAGGCTTGT |
| CotAD_28189 | TGGTGGATCAAACTCCCTGG | ACAAATTTAAGCTCTCTGTTGCT |
| CotAD_02795 | CAAGGCCTACTCCATGCGTC | TTTCGGCTAACCACGCACA |

**Additional file 8: Table S6. A list of primers used for SSCP, HRM and qRT-PCR analyses**
